# Effect of Age and Sex on Lower Extremity Power Production Capacity Throughout the Lifespan Based on 30 217 Finnish Participant Data

**DOI:** 10.1111/sms.70322

**Published:** 2026-06-18

**Authors:** Kaisa Koivunen, Eija K. Laakkonen, Juha P. Ahtiainen, Simon Walker

**Affiliations:** ^1^ Faculty of Sport and Health Sciences and Gerontology Research Center University of Jyväskylä Jyväskylä Finland; ^2^ Faculty of Sport and Health Sciences, NeuroMuscular Research Center University of Jyväskylä Jyväskylä Finland

**Keywords:** countermovement jump, life course, muscular fitness, peak power, physical performance

## Abstract

This study examined lower‐extremity power using countermovement jump (CMJ) height and estimated peak power across the lifespan with five Finnish datasets. The pooled dataset included 24 804 males and 5413 females aged 6–75 years. CMJ height (cm) was measured on a contact mat from flight time(s), and peak power (W) was estimated using equations for adults and children and expressed also relative to body mass (W/kg). Five‐year age groups were formed for mean comparison. CMJ height and peak power increased throughout childhood and adolescence. From ages 11 to 15, males exhibited greater CMJ height and peak power than females. Based on mean values, CMJ height peaked at ages 16–20 and peak power at 21–25 in males, while both peaked at 21–25 in females. Males maintained their level until 26–30 years, whereas females showed a decline after the peak. Segmented regression models indicated earlier peaks for CMJ height (18 years in males, 17 in females) and slightly later peaks for peak power (21 years in males, 19 in females). Before the peaking age, annual relative increases calculated over 11–15 years were 5.6% versus 3.1% for CMJ height and 4.7%–7.6% vs. 4.0%–7.3% for peak power (males vs. females). After the peaking age, annual declines calculated over 52–58 years were 0.9% for CMJ height and 0.3%–0.7% for peak power in both sexes. The results provide insights into life‐course patterns and the transitional period of late adolescence and early adulthood, when jumping performance peaks and evidence is limited.

## 1 Introduction

Lower extremity power, that is, the ability to exert force rapidly, is an important marker of athletic capacity as well as health and functional ability at different ages. In childhood and youth, muscle power is associated with gross motor skills [1] and health‐related fitness, such as bone strength [2] and cardio‐metabolic risk factors [3]. After peaking in youth or adulthood, aging leads to a decline in power, which is likely to occur earlier and to a greater extent than strength and muscle mass due to reduced motor unit discharge rates and selective atrophy of type II muscle fibers at a younger age [4] that accompany reduced physical activity [5]. Importantly, previous research suggests that power plays an important role in predicting functional loss in later life. For example, Foldvari et al. [6] found that among several physiological and muscle parameters, lower extremity power was the strongest predictor of self‐reported functional status in females aged 70 or over. Also, Bean et al. [7] reported a 2–3‐fold greater risk of low power than low strength with mobility limitations in males and females aged 65 or older.

Vertical jump performance tests, such as the countermovement jump (CMJ), are widely used, simple, and reliable measures of lower extremity power in different age groups [8, 9]. Although previous research has reported an earlier and faster decline in power compared to strength [10], little is known about the specific patterns of the changes in jumping performance across the entire lifespan, which could provide valuable insights into critical periods of development and the onset of deterioration. This knowledge could offer a strategic window for interventions aimed at maximizing peak performance and mitigating later declines. Most previous studies investigating age‐related changes in jumping performance have focused on children and adolescents [11, 12, 13] or on the adult population [14, 15, 16, 17] in isolation. Based on studies in younger populations, males tend to show a linear increase throughout youth, whereas in females, the curve flattens during early adolescence, coinciding with puberty. However, studying jump performance across the full lifespan is essential to identify the timing of the inflection points, for example, whether females experience further improvement after the previously observed plateau and when the turning point occurs in males.

Over 40 years ago, Bosco and Komi [18] reported jumping performance results in a convenience sample of Finnish individuals ranging from 4 to 73 years (*n* = 226), showing that lower extremity power reached its peak in males in their early 20s and in females earlier in adolescence. It is possible that jumping performance and its profile throughout the lifespan have changed in the current age cohorts. Our previous research suggests that the physical capacity of current older adults has improved compared to their same‐aged counterparts three decades earlier, likely due to more favorable life course exposures, such as improved education and health care, better nutrition, and increased physical activity [19]. In contrast, recent trends in younger cohorts point to a decline in physical activity [20] and physical functioning, particularly in jump performance [21]. These trends suggest that age‐related profiles of lower extremity power may have changed. Thus, the purpose of this study was to determine lower extremity power, measured by CMJ and estimated peak power, for both sexes in the current cohort across the life span.

## 2 Materials and Methods

We report cross‐sectional analyses using five Finnish datasets, all of which included CMJ tests conducted with comparable methods: Events dataset, Football/Floorball dataset, ERMA/EsmiRs cohort study, TraDeRe study, and FERTILE/ExHRT study.

### 2.1 Events Dataset

A convenience sample of 446 males and females aged from 6 to 75 years was measured in three public events in Finland: (1) The European Researchers' Night event in 2018 at the University of Jyväskylä campus (*n* = 151), (2) the Finnish Athletics Championships (Finnish: Kalevan kisat 2020) in 2018 (*n* = 165), held in the city of Jyväskylä, and (3) the Nordic Business Forum in 2016 held in Helsinki (*n* = 130). Inclusion criteria were willingness to take part, ability to consent, and ability to perform maximal CMJ safely [22]. Vertical jump performance was performed in everyday clothing. Two outliers, who upon discussion had clear ongoing athletic training in power sports, with CMJ results greater than 60 cm were excluded from the analyses. This resulted in 238 males and 206 females from this dataset included in the analytical sample.

### 2.2 Football/Floorball Dataset

The dataset consists of 8–20 years old male (*n* = 24 426) and 8–19 years old female (*n* = 4048) football and floorball players whose physical performance was tested in the Eerikkilä Sport & Outdoor Resort, Finland between the years 2017 and 2023. The tests were planned and conducted in collaboration with the Football Association of Finland and the Finnish Floorball Federation. Vertical jump performance was performed in sports clothing (i.e., shorts, t‐shirts etc.). This dataset was a non‐scientific study, performed as part of the agreed operations of each Federation who track the performance levels of their high‐performing junior athletes. Two outliers having < 10 cm CMJ results were excluded from the analyses, which resulted in 24 424 males and 4048 females included in the analytical sample.

### 2.3 ERMA and EsmiRs Study

Estrogenic Regulation of Muscle Apoptosis (ERMA) [23, 24] and its 4‐year follow‐up study Estrogen, MicroRNAs and the Risk of Metabolic Dysfunction (EsmiRs) are population‐based cohort studies, originally designed to investigate menopause and health in middle‐aged females. The ERMA sample was drawn from the Finnish Population Information System in 2014 comprising females aged 47–55 years, living in the city of Jyväskylä and neighboring municipalities in Finland (*n* = 3064). Exclusion criteria included a self‐reported body mass index (BMI) > 35 kg/m^2^, being currently pregnant or lactating, medical conditions or medications affecting ovarian function or systemic estrogen levels, and chronic diseases or medications seriously affecting muscle function. Data collection (ERMA in 2015–2016 and EsmiRs in 2018–2020) included questionnaires, biological sampling (blood samples and muscle biopsies for a subset of participants), and laboratory measurements. In total, 914 completed physiological and psychological measurements [23]. CMJ data was available for 825 participants in the ERMA baseline data collection. If the participant's CMJ result was not available at baseline, we used follow‐up data, where available, when the participants were approximately 4 years older (*n* = 45). This resulted in a total of 870 middle‐aged females for the analytical sample.

### 2.4 TraDeRe Study

Resistance TRAining, DEtraining, and REtraining (TraDeRe) study aims to identify the determinants of individual variation in resistance training responses. The study utilized a convenience sample of healthy participants who met the inclusion criteria: age within the range 18–50 years, no regular resistance training history, not participating in systematic endurance‐type training. Exclusion criteria were a history of medication that could affect exercise responses, use of creatine, any acute or chronic illness affecting cardiovascular, respiratory, musculoskeletal, and/or endocrine function, any other condition that may limit the ability to perform resistance training and testing (e.g., uncontrolled hypertension, diabetes, arthritic conditions, and neuromuscular complications), or blood‐borne diseases, diseases, and medication affecting blood clotting, allergies to anesthetic drugs, and severe psychological disorders. In this study, we used CMJ measurements conducted at study baseline (*n* = 326), including 142 males and 184 females.

### 2.5 FERTILE/ExHRT Study

The FERTILE women's cohort and 10‐year follow‐up study of the Exercise and Hormone Replacement Therapy (ExHRT) trial (FERTILE/ExHRT) comprises two datasets collected concurrently in 2007 [25]. The FERTILE women's cohort includes premenopausal women aged 30–40 years with regular menstrual cycles and not using hormonal contraceptives. Participants were screened among the 2000 women (39% of the entire cohort) randomly drawn from the age cohort living in the city of Jyväskylä. Exclusion criteria included irregular menstrual cycles, breastfeeding, planned pregnancy, current or past use of hormonal contraceptives within the previous 5 years, and health‐related contraindications. In total, 62 women participated in the laboratory assessments, which were conducted during the first 5 days of the menstrual cycle, and 61 had CMJ results available for the current study.

The ExHRT cohort includes postmenopausal women aged 61–68 years who had participated in ExHRT‐trial 10 years earlier. An invitation to participate was sent to all 80 women who took part in the baseline measurements of the trial; two were deceased, eight did not respond, and 23 were unwilling or unable to participate. Consequently, 47 women participated in the 10‐year follow‐up, and 44 had CMJ results available for the current study.

### 2.6 Test Procedures

In all studies, CMJ was measured on a contact mat recording flight time to the nearest 1/1000th of a second. Jump height (cm) was calculated from flight time: (g × *t* ^2^) ÷ 8 × 100 [26].

In the Events study, the participants were instructed on the CMJ technique verbally, and the tester demonstrated the technique with a sub‐maximal jump [22]. The participants were instructed to keep their hands on their hips while maintaining their gaze on the horizon when performing the jump to minimize the upper body contribution. The participants were asked to jump as high as possible with maximal effort. Two to four practice jumps were allowed, after which the participants completed the maximal effort testing. A short rest (30 s) was allowed between the jumps, and maximal effort jumps were repeated until the two best jumps produced results within 5% of each other. This procedure resulted in three to six maximal test jumps, and the best result was used in the analyses. Within‐session trial‐to‐trial variability of CMJ height was tested using the Events dataset. Mean standard error of measurement (SEM) ranged from approximately 0.5 to 1 cm, and mean coefficient of variation (CV) from 3.6% to 8.8%, indicating good measurement stability across different age groups.

In the ERMA/EsmiRs, the FERTILE/ExHRT and the TraDeRe studies, the instructions to participants were the same as in the Events study, but three to five maximal efforts were performed, and the highest value was used in the analysis. In the Football/Floorball sample, the participants performed three maximal efforts, following similar instructions, and the highest value was used in the analysis. All CMJ assessments were conducted by experienced testers following a standardized protocol, and trials were visually monitored and rejected if the instructed jumping technique was not maintained (e.g., knee flexion during the landing).

We estimated *peak power* (W) to study the rate at which an individual can generate force during a jump relative to their body weight. Peak power was first calculated in watts (W) and subsequently scaled to body mass (W/kg). The validated equations developed for the adult population may result in biased estimates for children due to children's lower body mass compared to adults. Therefore, we used different equations tailored to the adult and pediatric population to enhance the accuracy of the estimates. For participants of 18 years and over, peak power was calculated by the following equation of Sayers et al. [27]: Power (W) = 48.3*CMJ height (cm) + 50.1*body mass (kg) – 1890. For participants < 18 years, we used the equation by Gomez‐Bruton et al. [28] validated in children and adolescents aged 9–17 years: Power (W) = 54.2 × CMJ (cm) + 34.4 × body mass (kg) – 1520.4.

### 2.7 Other Variables

Other variables included age, sex, height (cm), and body mass (kg). In the Events dataset, the participants self‐reported their age, height, body mass, and sex. The participants of the football/floorball sample, age and sex were drawn from the sports association registration database, and height and body mass were measured using calibrated scalesat the Eerikkilä testing center by their staff. ERMA/EsmiRs and FERTILE/ExHRT samples included only females, and their age was derived from the Digital and Population Data Services Agency. Height and body mass were measured using digital scales following an overnight fast, wearing undergarments in the laboratory. In TraDeRe, age and sex were self‐reported, and height and body mass were measured similarly as in the ERMA/EsmiR study.

### 2.8 Statistical Analysis

Statistical analyses were performed with SPSS statistical software (Chicago, IL) version 28 and R environment (version 4.4.0). The analyses were conducted separately for males and females. Descriptive data are presented as means and standard deviations (SD) by 5‐year age groups. Differences in CMJ and estimated peak power between males and females in age‐matched groups were evaluated using *t*‐tests. The level of significance was set at *α* = 0.05 using two‐tailed testing. The *ggplot2* (v. 3.5.1) R package [29] was used for the visualization of the data.

We used one‐way analysis of variance (ANOVA) to examine differences in CMJ height and estimated peak power between age groups. Tukey's post hoc test was performed to determine which age groups differed significantly from the age group with the highest mean performance (reference group).

To examine non‐linear age‐related changes in performance, we examined the fit of second‐order (quadratic), third‐order (cubic) polynomial, and segmented linear regression models separately for males and females. The models included age as a continuous predictor and countermovement jump (CMJ) height and estimated peak power as outcome variables. To identify the most appropriate age breakpoint in the segmented regression models, we systematically tested all integer breakpoints between ages 10 and 40. For each candidate cut‐point, we fitted a two‐segment linear model and selected the optimal breakpoint based on the model with the lowest Akaike information criterion (AIC). The breakpoint optimization and modeling were performed separately for males and females.

Finally, model fit in terms of *R* ^2^ and AIC of the segmented regression models with the statistically optimal age breakpoint was compared with those obtained from the respective quadratic and cubic regression models (Table S6). In both sexes and for both CMJ height and peak power output, the segmented models yielded higher *R* ^2^ and lower AIC values than quadratic or cubic models, indicating superior model fit. Therefore, we proceeded with segmented modeling for all further visualizations and interpretation. The annual relative rates of change in CMJ height and peak power output were estimated from the slope coefficients before and after the breakpoint in the segmented regression models.

## 3 Results

**FIGURE 1:**
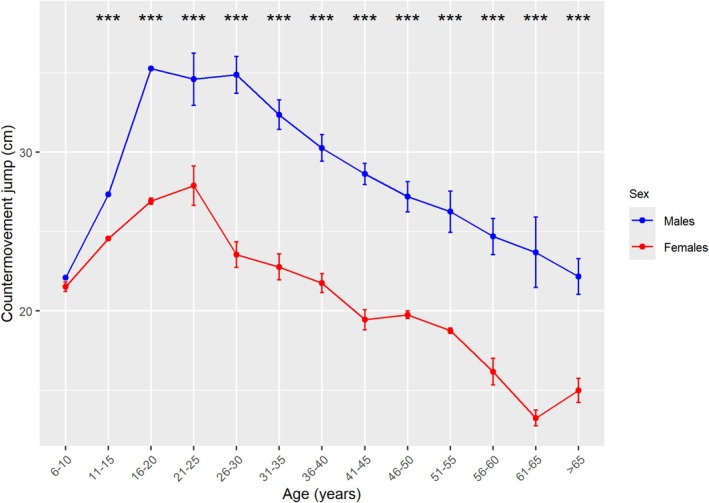
The average countermovement jump (CMJ) height with standard errors (SE) across 5‐year age groups in males and females. Statistical significance of sex differences across age groups is based on *p*‐values from independent *t*‐tests: ***p < 0.001, and no asterisk indicates *p* ≥ 0.05.

**FIGURE 2:**
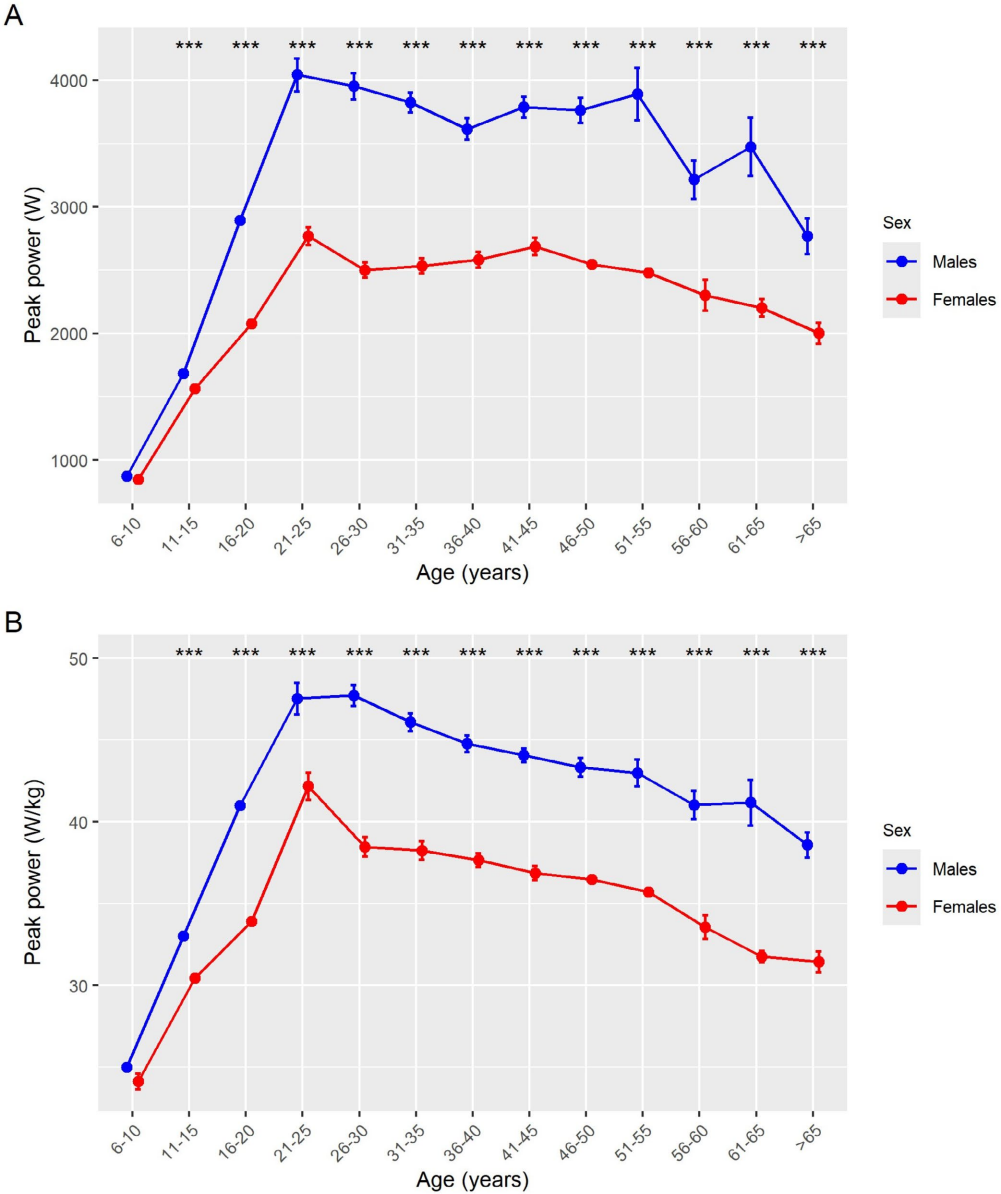
The average estimated peak power performance across 5‐year age groups in males and females: (A) peak power (W) and (B) peak power relative to body mass (W/kg), both presented with standard errors (SE). For participants < 18 years, estimated with the equation of Gomez‐Bruton et al. [28]: Power (W) = 54.2 × CMJ (cm) + 34.4 × body mass (kg) – 1520.4 and for participants of 18 years and over, with the equation of Sayers et al. [27]: Power (W) = 48.3* CMJ height (cm) + 50.1*body mass (kg) − 1890. Statistical significance of sex differences across age groups is based on *p*‐values from independent *t*‐tests: ***p < 0.001, and no asterisk indicates *p* ≥ 0.05.

The pooled dataset comprised 24 804 males and 5413 females, aged 6–75 years. Participant characteristics, including CMJ height and estimated peak power across 5‐year age groups are shown in Tables 1 and 2 for males and females, respectively. Dataset‐specific descriptive statistics are provided in Tables S1–S5 and Figures S1–S3. Children in the 6–10‐year age group showed similar performance in CMJ height and peak power (Figures 1 and 2). From the 11 to 15‐year age group onward, males demonstrated superior CMJ height compared to females, along with higher peak power across age groups from 11 to 15 up to > 65 years (*p* < 0.05).

**TABLE 1:** Descriptive statistics of the pooled datasets according to age groups in males (*n* = 24 804).

Age group	*n*	Age (years)	Height (cm)	Body mass (kg)	CMJ (cm)	Peak power (W)^a^	Peak power (W/kg)
		Mean (SD)	Mean (SD)	Mean (SD)	Mean (SD)	Mean (SD)	Mean (SD)
6–10	1205	9.8 (0.4)	142.3 (6.1)	34.7 (5.3)	**22.1 (3.4**)	**872 (243)**	**25.0 (5.4)**
11–15	21 492	13.1 (1.2)	161.5 (11.7)	50.1 (11.6)	**27.3 (5.3)**	**1684 (580)**	**33.0 (5.9)**
16–20	1764	16.7 (0.9)	179.3 (6.8)	70.5 (8.8)	35.3 (4.7)^ref^	**2893 (512)**	**41.0 (4.9)**
21–25	39	23.2 (1.4)	182.2 (6.3)	85.1 (14.3)	34.6 (10.3)	4044 (819)^ref^	47.5 (5.9)
26–30	54	28.1 (1.4)	179.5 (7.1)	83.0 (15.2)	34.9 (8.5)	3953 (750)	47.7 (4.7)^ref^
31–35	71	33.1 (1.5)	180.4 (6.7)	82.9 (10.8)	**32.4 (7.8)**	3824 (659)	46.1 (4.6)
36–40	50	38.0 (1.4)	177.5 (6.1)	80.7 (11.7)	**30.3 (5.9)**	**3615 (595)**	44.8 (3.7)
41–45	63	43.2 (1.4)	180.5 (7.0)	85.8 (12.5)	**28.6 (5.3)**	3788 (652)	**44.1 (3.2)**
46–50	35	47.5 (1.4)	180.8 (6.5)	86.6 (11.0)	**27.2 (5.7)**	3764 (594)	**43.3 (3.4)**
51–55	10	53.1 (1.4)	182.2 (6.7)	90.1 (10.2)	**26.2 (4.1)**	3891 (651)	43.0 (2.6)
56–60	10	57.3 (1.6)	176.7 (5.7)	78.1 (8.2)	**24.7 (3.6)**	**3215 (480)**	**41.0 (2.8)**
61–65	4	63.0 (1.6)	177.8 (3.3)	84.3 (7.3)	**23.7 (4.4)**	3475 (460)	41.2 (2.8)
> 65	7	69.6 (2.6)	176.0 (7.0)	71.6 (7.5)	**22.1 (3.0)**	**2770 (373)**	**38.6 (2.0)**

*Note:* Bolded values indicate a statistically significant difference compared to the reference group (ref; the age group with the highest performance), analyzed by ANOVA.

Abbreviations: cm, centimeter; *n*, number of subjects; SD, standard deviation; W, watts.

^a^ Peak power estimated for participants < 18 years, with the equation of Gomez‐Bruton et al. [28]: Power (W) = 54.2 × CMJ (cm) + 34.4 × body mass (kg) – 1520.4 and for participants of 18 years and over, with the equation of Sayers et al. [27]: Power (W) = 48.3*CMJ height (cm) + 50.1*body mass (kg) − 1890.

**TABLE 2:** Descriptive statistics of the pooled datasets according to age groups in females (*n* = 5413).

Age group	*n*	Age (years)	Height (cm)	Body mass (kg)	CMJ (cm)	Peak power (W)^a^	Peak power (W/kg)
		Mean (SD)	Mean (SD)	Mean (SD)	Mean (SD)	Mean (SD)	Mean (SD)
6–10	184	9.7 (0.8)	142.0 (6.5)	34.9 (5.8)	**21.5 (4.1)**	**846 (272)**	**24.1 (6.5)**
11–15	3496	13.2 (1.2)	159.7 (8.2)	51.0 (10.1)	**24.5 (4.0)**	**1563 (416)**	**30.4 (4.7)**
16–20	402	16.4 (0.8)	166.3 (6.4)	61.3 (8.8)	26.9 (4.0)	**2077 (372)**	**33.9 (4.0)**
21–25	50	23.2 (1.2)	167.8 (6.6)	66.1 (11.6)	27.9 (8.8)^ref^	2767 (499)^ref^	42.2 (5.9)^ref^
26–30	64	28.3 (1.4)	166.0 (6.1)	64.9 (10.0)	**23.5 (6.4)**	**2501 (490)**	**38.5(4.7)**
31–35	75	33.2 (1.3)	165.8 (6.1)	66.4 (10.0)	**22.8 (7.0)**	2538 (515)	**38.3 (5.0)**
36–40	102	38.3 (1.5)	165.2 (5.8)	68.3 (13.4)	**21.8 (6.0)**	2582 (627)	**37.7 (4.3)**
41–45	70	42.9 (1.5)	167.9 (6.1)	72.6 (11.9)	**19.5 (5.3)**	2688 (557)	**36.9 (3.7)**
46–50	380	49.5 (1.0)	166.0 (5.7)	65.5 (10.3)	**19.8 (4.6)**	**2546 (500)**	**36.5 (3.3)**
51–55	502	53.0 (1.2)	165.1 (5.7)	69.2 (10.8)	**18.7 (3.9)**	**2480 (495)**	**35.7 (2.8)**
56–60	17	57.2 (1.0)	165.9 (5.7)	68.1 (10.6)	**16.2 (3.5)**	**2302 (508)**	**33.6 (3.0)**
61–65	45	63.0 (1.3)	163.5 (5.4)	68.9 (10.7)	**13.3 (3.2)**	**2203 (458)**	**31.8 (2.3)**
> 65	26	67.6 (2.2)	161.7 (7.3)	63.2 (9.2)	**15.0 (3.9)**	**2003 (420)**	**31.5 (3.3)**

*Note:* Bolded values indicate a statistically significant difference compared to the reference group (ref; the age group with the highest performance), analyzed by ANOVA.

Abbreviations: *n*, number of subjects; W, watt.

^a^ Peak power estimated for participants < 18 years, with the equation of Gomez‐Bruton et al. [28]: Power (W) = 54.2 × CMJ (cm) + 34.4 × body mass (kg) – 1520.4 and for participants of 18 years and over, with the equation of Sayers et al. [27]: Power (W) = 48.3*CMJ height (cm) + 50.1*body mass (kg) − 1890.

**FIGURE 3:**
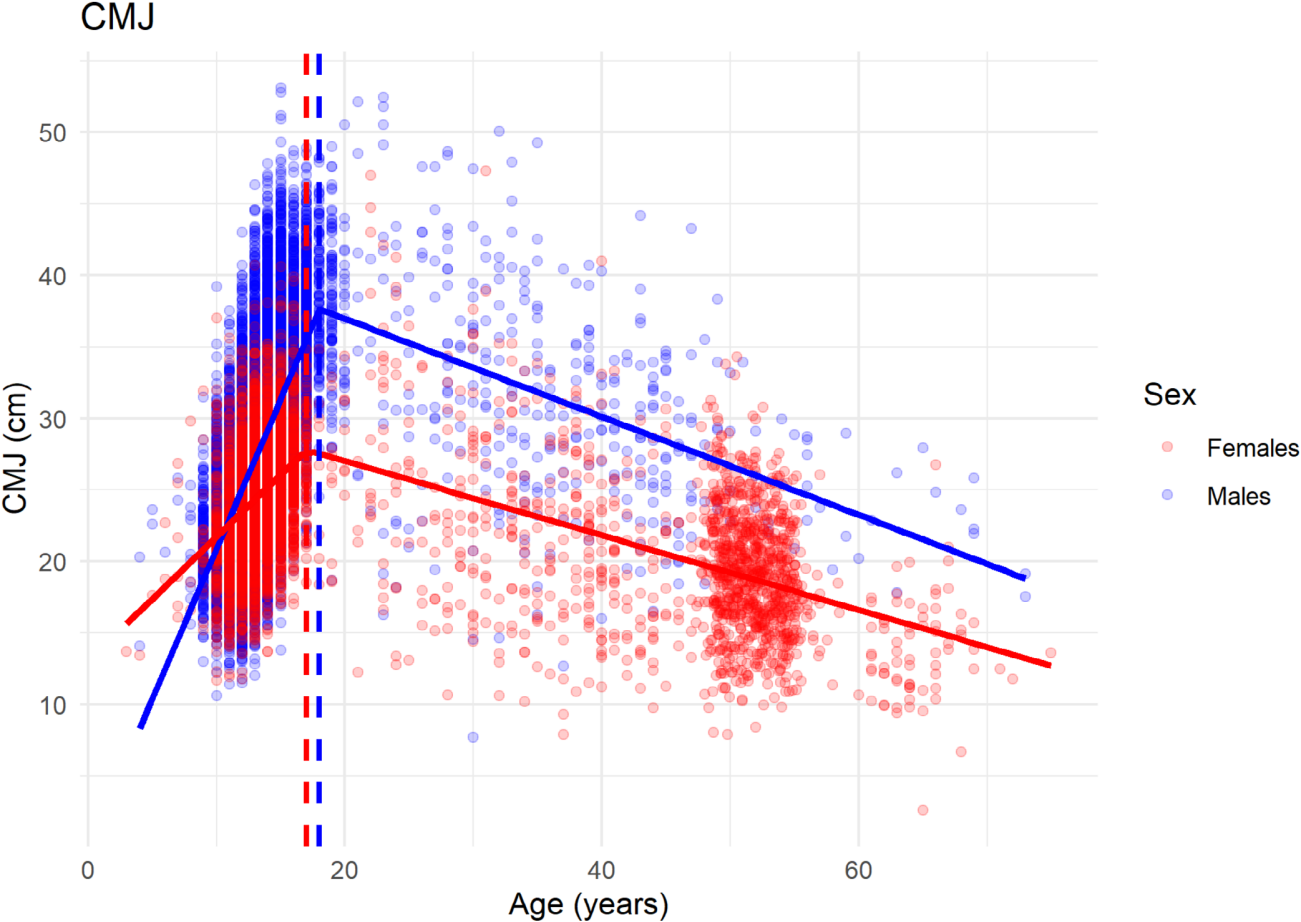
A segmented regression model of the countermovement (CMJ) jump height across age groups. The peaking age is 18 years in males and 17 years in females.

**FIGURE 4:**
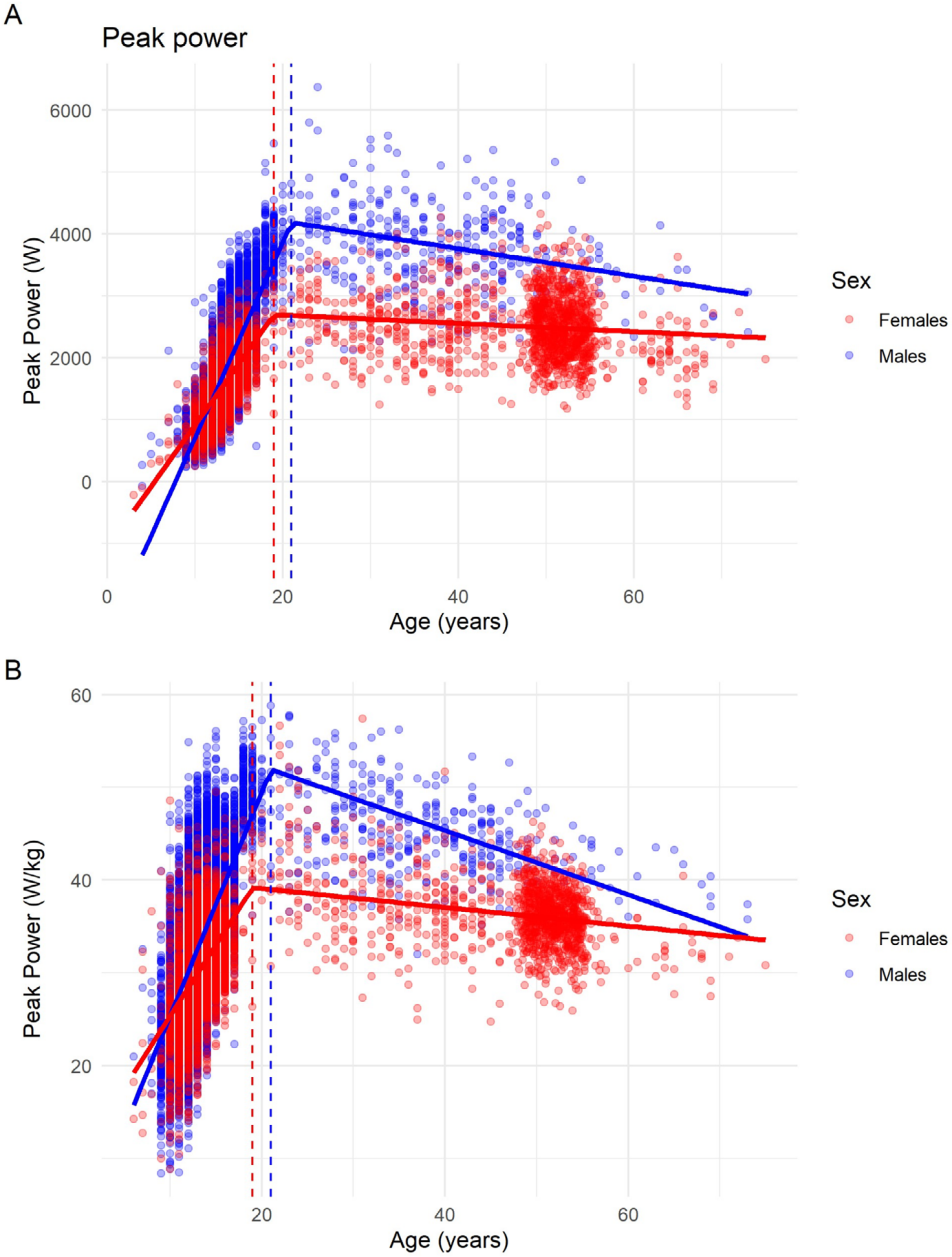
A segmented regression model of estimated peak power across age groups: (A) peak power (W) and (B) peak power relative to body mass (W/kg). Peak power was estimated using participants' countermovement jump (CMJ) height and body mass. For participants < 18 years, estimated with the equation of Gomez‐Bruton et al. [28]: Power (W) = 54.2 × CMJ (cm) + 34.4 × body mass (kg) – 1520.4 and for participants of 18 years and over, with the equation of Sayers et al. [27]: Power (W) = 48.3* CMJ height (cm) + 50.1*body mass (kg) − 1890. The peaking age 21 years in males and 19 years in females.

As expected, both CMJ height and peak power increased throughout childhood and adolescence. Based on the mean values in the 5‐year age groups, in males, CMJ height peaked at 16–20 years and estimated peak power at 21–25 years (Figures 1 and 2). In females, both the average CMJ height and estimated peak power reached their maximum at the age of 21–25. When estimated using segmented regression models, CMJ height peaked earlier, at 18 years in males and at 17 years in females, while peak power (both W and W/kg) reached its maximum later, at 21 years in males and 19 years in females (Figures 3 and 4).

The rate of increase in estimated peak power was steeper than that of CMJ height in both sexes, while its deterioration after the peaking age was slower. Additionally, the increase in CMJ height was more pronounced in males, while the subsequent decline was similar between sexes; changes in peak power were broadly comparable between sexes. Based on the segmented regression models, the average annual relative change before the peaking age in CMJ height was 5.6% in males and 3.1% in females (calculated over 12 and 11 years, respectively). After peaking, CMJ height declined at a rate of 0.9% per year in both sexes (calculated over 55 and 58 years, respectively). For peak power, the average annual relative change of increase before the peak was observed was 7.6% in males and 7.3% in females (calculated over 15 and 13 years, respectively), and 4.7% and 4.0% when expressed relative to body mass (W/kg). After the peaking age, peak power declined at a rate of 0.5% in males and 0.3% in females, and at a rate of 0.7% and 0.3% for W/kg (calculated over 52 and 56 years, respectively).

In the segmented regression model, age accounted for 38% of the variance in CMJ height in males (*R* ^2^ = 0.38) and 30% in females (*R* ^2^ = 0.30). For peak power, the model explained 67% of the variance in males and 63% in females, and 44% and 40% for peak power relative to body mass (W/kg), respectively (Table S6).

## 4 Discussion

In the present study, lower extremity power, assessed by CMJ height and estimated peak power, reaches its maximum in late adolescence or early adulthood, around age 20 in males and slightly earlier in females, followed by a gradual decline. The pattern of age‐related changes differs between CMJ height and estimated peak power. Peak power, which combines both jump height and body mass, tended to peak slightly later and decline more slowly than jump height, as increases in body mass during adulthood partly offset reductions in jump height. Given the technical demands of the CMJ, which require coordinated force production and motor control, age‐related differences may reflect not only muscular power but broader neuromuscular performance relevant to everyday movements. The early appearance of changes in jump height and peak power in these analyses suggests that power‐based tests may be particularly sensitive to age‐related neuromuscular changes, in line with previous findings [30].

Our findings align with developmental patterns observed in earlier studies, which have shown that CMJ height increases steadily throughout childhood and adolescence in males, continuing into their 20s. Previous studies on children and adolescents have shown that CMJ in females typically peaks and/or plateaus during adolescence [31, 32], which aligns with our segmented regression analysis indicating peak CMJ height at age 17 and peak power at age 19. However, comparisons in our mean data show that both CMJ height and estimated peak power in females continued to increase beyond adolescence, reaching their highest values in the 21–25‐year age groups. This differs from earlier reports suggesting minimal gains in CMJ height after adolescence [31, 32]. It is important to note that the 21–25‐year‐old female group included only 50 participants and showed substantial variability in performance, whereas the younger age groups were dominated by athletes. Therefore, the exact peaks of the present study should be interpreted with caution and further examined in a larger and more representative population‐based sample. Nevertheless, these results highlight a critical window during youth and early adulthood for optimizing power output and neuromuscular health, which may have implications for performance and functional capacity later in life.

In our study, the rate of decline in absolute CMJ height and estimated peak power with age was more pronounced in males than in females; however, the relative rate of decline was comparable between the sexes, aligning with previous findings [33]. These sex differences in age‐related decline in absolute power are largely attributable to the persistent disparity in muscle mass between men and women across the lifespan [34]. Due to generally lower absolute values, females may reach the “disability threshold” (the critical level of physical capacity below which mobility limitations are more likely to occur) earlier with aging [35]. However, evidence from female master athletes (> 55 years) indicates that women can outperform sedentary men of the same age, suggesting that regular training may mitigate the age‐related decline [36].

Our observations showed an annual decline of approximately 1% in CMJ height in both sexes and a decrease in peak power of 0.5%–0.7% in males and 0.3% in females after the peak performance age of approximately 20. The decline in CMJ height is consistent with the annual rates reported by Meulemans et al. [30], which ranged from 0.70% to 0.81% before age 60 and from 1.61% to 1.75% after age 60. Additionally, Westerståhl et al. [33] reported an average annual decrease of approximately 1% comparing best performance to performance at 63 years of age, using the Sargent jump test.

However, the observed rate of decline in peak power in our study was lower than previously reported. For example, Strotmeyer et al. [37], using force‐plate derived muscle power in men aged 77–101 years. Similarly, Riemann et al. [38] observed average annual declines in force‐plate–derived peak power of 1.7% in males and 0.9% in females between ages 40 and 75. Age‐related loss of muscle power is known to show relatively modest changes during early and mid‐adulthood, followed by a more pronounced decline at older ages [30]. The average annual decline observed in the present study (0.5%–0.7% in males and 0.3% in females) may, therefore, be attenuated because it spans a broad adult age range. In addition, differences in assessment methods, power estimation approaches, and population characteristics may contribute to the variation in reported rates of decline and may partly explain the slower decline observed.

To our knowledge, this is the first and most comprehensive analysis of age‐related changes in jump performance across the lifespan since the study by Bosco and Komi over four decades ago in Finnish participants [18], using a similar CMJ test procedure. Thus, comparable data covering the whole lifespan from other countries are lacking. When comparing the average CMJ results in age groups of these two studies, several differences emerge that may reflect birth cohort changes in physical performance. In males, the pattern of increasing CMJ height until the age of 20–25 with maximum estimates of ~35 cm was consistent with earlier findings, but unlike the earlier data, our data suggest that performance plateaued before declining. In contrast, females reached their performance maximum later than the previously reported peak at ages 9–12 years; while performance at those ages was comparable (~25 cm) to historical data, it continued to increase into early adulthood and ultimately exceeded the earlier maximum by ~3 cm. These findings propose that while maximum lower extremity power may have remained relatively stable in males, females have experienced improvements. For example, in Finland, teenage girls now participate in organized sports at higher rates than in previous decades, reflecting cultural shifts and equal access to sports opportunities [39]. Additionally, older adults around 60–70 years of age demonstrated CMJ heights approximately 5–10 cm higher than those reported in the past in both sexes, suggesting better maintenance of neuromuscular performance in later life. Ultimately, in both sexes, age‐related decline appears less steep, possibly reflecting better health, medical treatment and health care, fitness, and diet in the current population. These findings may be attributable to the reduced exposure of current cohorts to physically strenuous jobs, higher levels of education, improved economic status, greater awareness of the importance of physical functioning, and advancements in training practices [19]. Although direct comparisons of peak power output were not possible, the combination of higher body mass and maintained or improved CMJ height in the present study may imply enhanced physical capacity in current middle‐aged and older populations as heavier individuals with similar jump heights result in greater power values.

Pooling data from multiple sources that used similar jump testing protocols enabled us to examine jump performance across the entire lifespan, which is a key strength of our study. This provided valuable insights into the transitional phase between late adolescence and early adulthood, which is a period when jump performance typically peaks but has been relatively underrepresented in previous research. A notable limitation of the study is the lack of information on important covariates, particularly fat‐free mass and overall physical activity levels, as part of the data were derived from non‐scientific sources and public events. Further limitations of our study include the use of self‐reported body mass and height in the Events dataset, which may introduce measurement bias. In addition, estimated peak power was derived using the Sayers equation, which was originally validated in young adults and may not be optimal considering age‐related changes in body composition and neuromuscular function in older populations, and the results should be interpreted with caution. This highlights the need for future work to examine and possibly develop and validate peak power estimation equations across the entire adult lifespan. Alternative approaches for normalizing CMJ performance (e.g., ratio‐based or scaling methods) are also used in the literature. However, the estimation equations used here are widely applied and incorporate body mass as a predictor of peak power rather than assuming proportional scaling with body mass, being a pragmatic approach for large‐scale data collections where advanced instrumentation is not available.

Combining different datasets may introduce selection bias and influence absolute performance levels. For example, the inclusion of football and floorball players may have elevated performance levels among younger participants relative to the general population, which should be considered when interpreting absolute values. However, the inclusion of a large and heterogeneous sample may help reduce the relative influence of any single selected subgroup on the observed age‐related trajectories. When compared with existing normative data from England [13] and Colombia [32], our sample's vertical jump heights are broadly similar to those reported for school‐aged children and adolescents when measured without arm swing. This comparison suggests that our findings for children and adolescents do not overestimate jumping performance relative to the general population. Additionally, the unequal sample size between sexes in younger age groups, particularly the underrepresentation of females compared to males, should be acknowledged. This imbalance also highlights prevailing practices in Finland, where physical performance testing is conducted more systematically in males, for example, through mandatory military service in men and participation in major sports, which are most commonly practiced by men.

To conclude, our findings demonstrate life‐course patterns in lower extremity power in healthy individuals, with peak performance occurring in late adolescence and early adulthood, earlier in females than in males. As the divergence between males and females becomes apparent during this developmental stage, it may represent a potentially favorable window for targeting power training in females to maximize functional capacity. Comparison with Finnish data collected four decades ago further indicates higher performance levels and better preservation of neuromuscular function in later life among current birth cohorts.

## 5 Perspective

Knowledge of the timing and evolution of peak power performance is important for identifying windows of opportunity to optimize neuromuscular development and for designing strategies to preserve functional performance throughout adulthood and aging. Although countermovement jump tests have not been widely used in large‐scale population studies or national testing programs, they show promise as sensitive indicators of functional capacity and age‐related neuromuscular changes, particularly during development. Siglinsky et al. [16] reported that jump height had a stronger association with age than gait speed, grip strength, or chair rise performance in adults aged 27–96 years, suggesting its ability to detect subtle changes potentially missed by traditional performance‐based tests. According to earlier studies, the CMJ test is a reliable and feasible method for assessing lower‐body power in children and adults [8, 9], and its simplicity supports repeated measurements across diverse populations, including broader assessment of physical performance in younger females who are underrepresented compared with males in current national programs. Future use of wearable sensors [22] or AI‐driven smartphone applications [40] to measure jump height could enable wider application without specialized equipment, helping to implement this test into national or large‐scale testing programs.

## Funding

The data for the various cohorts were collected through funding provided by the Research Council of Finland to Eija K Laakkonen (#275323, #314181, #335249), Simon Walker (#350528), and Juha Ahtiainen (#357185).

## Ethics Statement

For the Events data collection, the ethics committee of the University of Jyväskylä declared that the study did not require an ethical statement because the data collection was completely anonymous, and the study was neither medical nor invasive. The study was conducted in accordance with the Declaration of Helsinki (2013) and followed the guidelines of the Finnish National Board on Research Integrity TENK. Either an informed written or verbal consent was obtained from all participants. Written informed consent was obtained from the guardians of the under‐aged participants (*n* = 45). The ERMA, EsmiRs and FERTILE/ExHRT studies were approved by the Ethics Committee of the Central Finland Health Care District (ERMA 8 U/2014, EsmiRs 9 U/2018 and FERTILE/ExHRT 5/2006). The TraDeRe study received ethical approval from the University of Jyväskylä's ethics committee (857/13.00.04.00/2021).

## Consent

The study protocols followed good clinical and scientific practice and the Declaration of Helsinki (2013). All study participants gave written informed consent. The athlete data of football and floorball players was not collected as part of a scientific study. Data collection at Eerikkilä Sport & Outdoor Resort, Finland, followed the established policies of the Football Association of Finland, the Finnish Floorball Federation, and the individual participating clubs, and consent was obtained through these procedures. All data were pseudonymized, and the research team had no access to personal identifying information.

## Conflicts of Interest

The authors declare no conflicts of interest.

## Supporting information


**Figure S1:** The relationship between countermovement jump (CMJ) and age for males and females across four datasets. The plot displays the mean CMJ values (in cm) ± standard error (SE).
**Figure S2:** The relationship between predicted peak power and age for males and females across five datasets. The plot displays the mean peak power values (W) ± standard errors (SE).
**Figure S3:** The relationship between predicted peak power scaled to body mass and age for males and females across five datasets. The plot displays mean peak power values (W/kg) ± standard errors (SE).
**Table S1:** Descriptive statistics of the Events dataset according to age groups and sex.
**Table S2:** Descriptive statistics of the Football/floorball dataset according to age groups and sex.
**Table S3:** Descriptive statistics of the TraDeRe dataset according to age groups and sex.
**Table S4:** Descriptive statistics of the ERMA/EsmiRs dataset according to age groups.
**Table S5:** Descriptive statistics of the FERTILE/ExHRT dataset according to age groups.
**Table S6:** Curvilinear regression analyses of the relationships between countermovement jump, peak power, and age.

## Data Availability

The data supporting the findings of this study are available upon reasonable request from the respective data holders: Event data: Simon Walker (simon.walker@jyu.fi); Estrogenic Regulation of Muscle Apoptosis (ERMA), Estrogen, MicroRNAs and the Risk of Metabolic Dysfunction (EsmiRs), and FERTILE women's cohort and 10‐year follow‐up study of the Exercise and Hormone Replacement Therapy (ExHRT) trial (FERTILE/ExHRT) data: Eija K. Laakkonen (eija.k.laakkonen@jyu.fi); TRAining, DEtraining, and REtraining (TraDeRe): Juha P. Ahtiainen (juha.ahtiainen@jyu.fi); Football/Floorball data: Elisa Hakamäki, Eerikkilä Sport & Outdoor Resort, Finland (elisa.hakamaki@eerikkila.fi).

## References

[1] J. L. Wang , S. H. Sun , and H. C. Lin , “Relationship of Quantitative Measures of Jumping Performance With Gross Motor Development in Typically Developed Preschool Children,” International Journal of Environmental Research and Public Health 19, no. 3 (2022): 1661, 10.3390/ijerph19031661. 35162684 PMC8835438

[2] K. F. Janz , E. M. Letuchy , T. L. Burns , S. L. Francis , and S. M. Levy , “Muscle Power Predicts Adolescent Bone Strength: Iowa Bone Development Study,” Medicine and Science in Sports and Exercise 47, no. 10 (2015): 2201–2206, 10.1249/MSS.0000000000000648. 25751769 PMC4549233

[3] M. Zaqout , N. Michels , K. Bammann , et al., “Influence of Physical Fitness on Cardio‐Metabolic Risk Factors in European Children. The IDEFICS Study,” International Journal of Obesity 40, no. 7 (2016): 1119–1125, 10.1038/ijo.2016.22. 26857382

[4] S. K. Hunter , H. M. Pereira , and K. G. Keenan , “The Aging Neuromuscular System and Motor Performance,” Journal of Applied Physiology 121, no. 4 (2016): 982–995, 10.1152/japplphysiol.00475.2016. 27516536 PMC5142309

[5] L. Hvid , P. Aagaard , L. Justesen , et al., “Effects of Aging on Muscle Mechanical Function and Muscle Fiber Morphology During Short‐Term Immobilization and Subsequent Retraining,” Journal of Applied Physiology 109, no. 6 (2010): 1628–1634, 10.1152/japplphysiol.00637.2010. 20864557

[6] M. Foldvari , M. Clark , L. C. Laviolette , et al., “Association of Muscle Power With Functional Status in Community‐Dwelling Elderly Women,” Journals of Gerontology. Series A, Biological Sciences and Medical Sciences 55, no. 4 (2000): M192–M199, 10.1093/gerona/55.4.M192. 10811148

[7] J. F. Bean , S. G. Leveille , D. K. Kiely , S. Bandinelli , J. M. Guralnik , and L. Ferrucci , “A Comparison of Leg Power and Leg Strength Within the InCHIANTI Study: Which Influences Mobility More?,” Journals of Gerontology. Series A, Biological Sciences and Medical Sciences 58, no. 8 (2003): M728–M733, 10.1093/gerona/58.8.M728. 12902531

[8] J. Rittweger , H. Schiessl , D. Felsenberg , and M. Runge , “Reproducibility of the Jumping Mechanography as a Test of Mechanical Power Output in Physically Competent Adult and Elderly Subjects,” Journal of the American Geriatrics Society 52, no. 1 (2004): 128–131, 10.1111/j.1532-5415.2004.52022.x. 14687327

[9] L. N. Veilleux and F. Rauch , “Reproducibility of Jumping Mechanography in Healthy Children and Adults,” Journal of Musculoskeletal & Neuronal Interactions 10, no. 4 (2010): 256–266. 21116062

[10] K. F. Reid and R. A. Fielding , “Skeletal Muscle Power: A Critical Determinant of Physical Functioning in Older Adults,” Exercise and Sport Sciences Reviews 40, no. 1 (2012): 4–12, 10.1097/JES.0b013e31823b5f13. 22016147 PMC3245773

[11] A. Focke , G. Strutzenberger , D. Jekauc , A. Worth , A. Woll , and H. Schwameder , “Effects of Age, Sex and Activity Level on Counter‐Movement Jump Performance in Children and Adolescents,” European Journal of Sport Science 13, no. 5 (2013): 518–526, 10.1080/17461391.2012.756069. 24050469

[12] Z. Sumnik , J. Matyskova , Z. Hlavka , L. Durdilova , O. Soucek , and D. Zemkova , “Reference Data for Jumping Mechanography in Healthy Children and Adolescents Aged 6‐18 Years,” Journal of Musculoskeletal & Neuronal Interactions 13, no. 3 (2013): 297–311. 23989251

[13] M. J. D. Taylor , D. Cohen , C. Voss , and G. R. H. Sandercock , “Vertical Jumping and Leg Power Normative Data for English School Children Aged 10–15 Years,” Journal of Sports Sciences 28, no. 8 (2010): 867–872, 10.1080/02640411003770212. 20496221

[14] R. Dietzel , U. Gast , T. Heine , D. Felsenberg , and G. Armbrecht , “Cross‐Sectional Assessment of Neuromuscular Function Using Mechanography in Women and Men Aged 20‐85 Years,” Journal of Musculoskeletal & Neuronal Interactions 13, no. 3 (2013): 312–319. 23989252

[15] Y. Dionyssiotis , “Assessment of Musculoskeletal System in Women With Jumping Mechanography,” International Journal of Women's Health 1 (2009): 113–118, 10.2147/IJWH.S5889. PMC2971710 21072281

[16] E. Siglinsky , D. Krueger , R. E. Ward , et al., “Effect of Age and Sex on Jumping Mechanography and Other Measures of Muscle Mass and Function,” Journal of Musculoskeletal & Neuronal Interactions 15, no. 4 (2015): 301–308. 26636275 PMC4784267

[17] S. Wiegmann , D. Felsenberg , G. Armbrecht , and R. Dietzel , “Longitudinal Changes in Muscle Power Compared to Muscle Strength and Mass,” Journal of Musculoskeletal & Neuronal Interactions 21, no. 1 (2021): 13–25. 33657752 PMC8020018

[18] C. Bosco and P. V. Komi , “Influence of Aging on the Mechanical Behavior of Leg Extensor Muscles,” European Journal of Applied Physiology 45, no. 2–3 (1980): 209–219, 10.1007/BF00421329. 7193130

[19] K. Koivunen , E. Sillanpää , M. Munukka , E. Portegijs , and T. Rantanen , “Cohort Differences in Maximal Physical Performance: A Comparison of 75‐ and 80‐Year‐Old Men and Women Born 28 Years Apart,” Journals of Gerontology. Series A, Biological Sciences and Medical Sciences 76, no. 7 (2021): 1251–1259, 10.1093/gerona/glaa224. 32886740

[20] S. A. Conger , L. P. Toth , C. Cretsinger , et al., “Time Trends in Physical Activity Using Wearable Devices: A Systematic Review and Meta‐Analysis of Studies From 1995 to 2017,” Medicine and Science in Sports and Exercise 54, no. 2 (2022): 288–298, 10.1249/MSS.0000000000002794. 34559725

[21] B. J. Fraser , L. Blizzard , G. R. Tomkinson , et al., “The Great Leap Backward: Changes in the Jumping Performance of Australian Children Aged 11−12‐Years Between 1985 and 2015,” Journal of Sports Sciences 37, no. 7 (2019): 748–754, 10.1080/02640414.2018.1523672. 30319026

[22] T. Rantalainen , T. Finni , and S. Walker , “Jump Height From Inertial Recordings: A Tutorial for a Sports Scientist,” Scandinavian Journal of Medicine & Science in Sports 30, no. 1 (2020): 38–45, 10.1111/sms.13546. 31483899

[23] E. Laakkonen , S. Sipilä , and V. Kovanen , “Data From Estrogenic Regulation of Muscle Apoptosis (ERMA) Study,” 2022, 10.17011/jyx/dataset/83491. PMC6110369 29738416

[24] V. Kovanen , P. Aukee , K. Kokko , et al., “Design and Protocol of Estrogenic Regulation of Muscle Apoptosis (ERMA) Study With 47 to 55‐Year‐Old Women's Cohort: Novel Results Show Menopause‐Related Differences in Blood Count,” Menopause 25, no. 9 (2018): 1020–1032, 10.1097/GME.0000000000001117. 29738416 PMC6110369

[25] E. Pöllänen , S. Sipilä , M. Alen , et al., “Differential Influence of Peripheral and Systemic Sex Steroids on Skeletal Muscle Quality in Pre‐ and Postmenopausal Women,” Aging Cell 10, no. 4 (2011): 650–660, 10.1111/j.1474-9726.2011.00701.x. 21388496

[26] C. Bosco , P. Luhtanen , and P. V. Komi , “A Simple Method for Measurement of Mechanical Power in Jumping,” European Journal of Applied Physiology 50, no. 2 (1983): 273–282, 10.1007/BF00422166. 6681758

[27] S. P. Sayers , D. V. Harackiewicz , E. A. Harman , P. N. Frykman , and M. T. Rosenstein , “Cross‐Validation of Three Jump Power Equations,” Medicine and Science in Sports and Exercise 31, no. 4 (1999): 572–577, 10.1097/00005768-199904000-00013. 10211854

[28] A. Gomez‐Bruton , L. Gabel , L. Nettlefold , H. Macdonald , D. Race , and H. McKay , “Estimation of Peak Muscle Power From a Countermovement Vertical Jump in Children and Adolescents,” Journal of Strength and Conditioning Research 33, no. 2 (2019): 390–398, 10.1519/JSC.0000000000002002. 28570492

[29] H. Wickham , W. Chang , L. Henry , et al., “ggplot2: Create Elegant Data Visualisations Using the Grammar of Graphics,” 2007:3.5.1, 10.32614/CRAN.package.ggplot2.

[30] L. Meulemans , J. Deboutte , J. Seghers , C. Delecluse , and E. Van Roie , “Age‐Related Differences Across the Adult Lifespan: A Comparison of Six Field Assessments of Physical Function,” Aging Clinical and Experimental Research 37, no. 1 (2025): 72, 10.1007/s40520-025-02965-1. 40055287 PMC11889021

[31] K. R. Laurson , F. Baptista , M. T. Mahar , G. J. Welk , and K. F. Janz , “Long Jump, Vertical Jump, and Vertical Jump Power Reference Curves for 10‐18 Year Olds,” Measurement in Physical Education and Exercise Science 26, no. 4 (2022): 306–314, 10.1080/1091367X.2021.2017291.

[32] R. Ramírez‐Vélez , J. E. Correa‐Bautista , F. Lobelo , E. L. Cadore , A. M. Alonso‐Martinez , and M. Izquierdo , “Vertical Jump and Leg Power Normative Data for Colombian Schoolchildren Aged 9–17.9 Years: The FUPRECOL Study,” Journal of Strength and Conditioning Research 31, no. 4 (2017): 990–998, 10.1519/JSC.0000000000001550. 28328716

[33] M. Westerståhl , G. Jörnåker , E. Jansson , et al., “Rise and Fall of Physical Capacity in a General Population: A 47‐Year Longitudinal Study,” Journal of Cachexia, Sarcopenia and Muscle 16, no. 6 (2025): e70134, 10.1002/jcsm.70134. 41243424 PMC12620399

[34] J. Alcazar , P. Aagaard , B. Haddock , et al., “Age‐ and Sex‐Specific Changes in Lower‐Limb Muscle Power Throughout the Lifespan,” Journals of Gerontology: Series A, Biological Sciences and Medical Sciences 75, no. 7 (2020): 1369–1378, 10.1093/gerona/glaa013. 31943003

[35] K. Koivunen , E. Portegijs , L. Karavirta , and T. Rantanen , “Comparing the Associations Between Muscle Strength, Walking Speed, and Mortality in Community‐Dwelling Older Adults of Two Birth Cohorts Born 28 Years Apart,” Geroscience 46, no. 2 (2024): 1575–1588, 10.1007/s11357-023-00925-z. 37656329 PMC10828148

[36] E. Fernández‐Peña , E. Formiglio , M. Gervasi , et al., “The Impact of Track and Field Training on Dynapenia: Gender Differences in Age‐Related Decline of Vertical Jump Performance Among Older Adults,” Frontiers in Aging 5 (2024): 1504789, 10.3389/fragi.2024.1504789. 39735687 PMC11672781

[37] E. S. Strotmeyer , M. E. Winger , J. A. Cauley , et al., “Normative Values of Muscle Power Using Force Plate Jump Tests in Men Aged 77–101 Years: The Osteoporotic Fractures in Men (MrOS) Study,” Journal of Nutrition, Health & Aging 22, no. 10 (2018): 1167–1175, 10.1007/s12603-018-1081-x. PMC8963464 30498822

[38] B. L. Riemann , M. Johnson , M. K. Helms , et al., “Countermovement Jump Peak Power Changes With Age in Masters Weightlifters,” Sports 12, no. 9 (2024): 259, 10.3390/sports12090259. 39330736 PMC11436148

[39] I. Lounassalo , T. Kukko , T. Suominen , et al., “Sociodemographic Determinants of Youth Sports Club Participation Across Two Generations: The Young Finns Study,” Journal of Public Health (2025), 10.1007/s10389-025-02500-6.

[40] P. T. Ríos‐Gallardo , L. E. Carranza‐García , C. Balsalobre‐Fernández , and S. Montalvo , “Reliability and Validity of an AI‐Driven Smartphone Application for Measuring Countermovement Jump Height: A Comparison With Force Platform, Infrared Optical Timing, and Manual Video Analysis,” Measurement in Physical Education and Exercise Science 30, no. 1 (2026): 73–86, 10.1080/1091367X.2025.2532391.

