# Supplementary material for: Effect of Age and Sex on Lower Extremity Power Production Capacity Throughout the Lifespan Based on 30 217 Finnish Participant Data
Source: Scand J Med Sci Sports. 2026 Jun 18;36(6):e70322. doi: 10.1111/sms.70322 (PMC13280190; doi:10.1111/sms.70322)
Supplement: Supplementary file 1 — Figure S1: The relationship between countermovement jump (CMJ) and age for males and females across four datasets. The plot displays the mean CMJ values (in cm) ± standard error (SE). Figure S2: The relationship between predicted peak power and age for males and females across five datasets. The plot displays the mean peak power values (W) ± standard errors (SE). Figure S3: The relationship between predicted peak power scaled to body mass and age for males and females across five datasets. The plot displays mean peak power values (W/kg) ± standard errors (SE). Table S1: Descriptive statistics of the Events dataset according to age groups and sex. Table S2: Descriptive statistics of the Football/floorball dataset according to age groups and sex. Table S3: Descriptive statistics of the TraDeRe dataset according to age groups and sex. Table S4: Descriptive statistics of the ERMA/EsmiRs dataset according to age groups. Table S5: Descriptive statistics of the FERTILE/ExHRT dataset according to age groups. Table S6: Curvilinear regression analyses of the relationships between countermovement jump, peak power, and age. [file SMS-36-e70322-s001.docx]

**Supplementary material**

Supplementary Table 1. Descriptive statistics of the Events dataset according to age groups and sex.

|  | **Events dataset – males (n=238)** | | | | | **Events dataset – females (n=206)** | | | | |
| --- | --- | --- | --- | --- | --- | --- | --- | --- | --- | --- |
| Age group | Nr of subjects | Age (years) | Height (cm) | Body mass (kg) | CMJ (cm) | Nr of subjects | Age | Height (cm) | Body mass (kg) | CMJ (cm) |
|  |  | Mean (sd) | Mean (sd) | Mean (sd) | Mean (sd) |  | Mean (sd) | Mean (sd) | Mean (sd) | Mean (sd) |
| 6-10 | 12 | 8.4 (1.2) | 146.2 (13.6) | 39.1 (13.2) | 22.6 (2.9) | 13 | 7.5 (1.2) | 139.4 (10.0) | 31.8 (7.2) | 23.6 (5.6) |
| 11-15 | 7 | 13.6 (1.9) | 170.9 (12.6) | 56.9 (11.5) | 35.9 (5.0) | 3 | 12.3 (2.3) | 159.0 (18.2) | 43.7 (16.1) | 19.5 (3.7) |
| 16-20 | 16 | 19.4 (0.6) | 181.3 (3.4) | 73.4 (7.6) | 34.2 (7.0) | 16 | 18.8 (1.2) | 169.9 (6.6) | 61.9 (7.7) | 26.5 (6.4) |
| 21-25 | 25 | 23.1 (1.4) | 182.8 (5.6) | 84.2 (13.3) | 40.7 (7.0) | 40 | 23.2 (1.1) | 167.8 (6.2) | 64.5 (8.6) | 30.0 (8.1) |
| 26-30 | 36 | 28.0 (1.1) | 179.6 (8.2) | 81.0 (12.1) | 37.7 (6.5) | 29 | 28.1 (1.3) | 166.7 (6.6) | 62.9 (8.7) | 25.2 (6.8) |
| 31-35 | 43 | 33.0 (1.4) | 182.2 (6.5) | 83.3 (10.1) | 35.8 (6.8) | 24 | 32.9 (1.3) | 167.5 (4.5) | 64.2 (9.1) | 25.5 (8.0) |
| 36-40 | 23 | 38.3 (1.2) | 178.5 (6.4) | 77.8 (9.2) | 33.6 (4.0) | 20 | 37.8 (1.3) | 166.5 (5.7) | 63.4 (9.0) | 26.1 (5.3) |
| 41-45 | 25 | 43.2 (1.6) | 180.4 (6.9) | 85.6 (10.3) | 32.3 (4.6) | 17 | 43.4 (1.7) | 167.1 (5.8) | 69.5 (11.1) | 23.6 (5.1) |
| 46-50 | 20 | 48.0 (1.5) | 182.6 (5.4) | 87.1 (8.1) | 30.0 (5.3) | 7 | 47.9 (2.0) | 167.9 (5.3) | 62.9 (10.6) | 20.5 (3.9) |
| 51-55 | 10 | 53.1 (1.4) | 182.2 (6.7) | 90.1 (10.2) | 26.2 (4.1) | 5 | 52.8 (0.8) | 166.2 (2.3) | 67.6 (7.8) | 19.8 (4.7) |
| 56-60 | 10 | 57.3 (1.6) | 176.7 (5.7) | 78.1 (8.2) | 24.7 (3.6) | 5 | 57.4 (1.5) | 167.8 (4.4) | 62.6 (6.5) | 15.2 (2.9) |
| 61-65 | 4 | 63.0 (1.6) | 177.8 (3.3) | 84.3 (7.3) | 23.7 (4.4) | 13 | 63.3 (1.5) | 166.2 (5.9) | 66.3 (9.6) | 14.4 (2.1) |
| >65 | 7 | 69.6 (2.6) | 176.0 (7.0) | 71.6 (7.5) | 22.1 (3.0) | 14 | 68.7 (2.5) | 164.5 (6.6) | 64.0 (8.7) | 16.1 (4.1) |

*Notes.* CMJ = Countermovement jump

Supplementary Table 2. Descriptive statistics of the Football/floorball dataset according to age groups and sex.

|  | **Football/floorball dataset – males** (n=24 424) | | | | | **Football/floorball dataset – females** (n=4 048) | | | | |
| --- | --- | --- | --- | --- | --- | --- | --- | --- | --- | --- |
| Age group | Nr of subjects | Age (years) | Height (cm) | Body mass (kg) | CMJ (cm) | Nr of subjects | Age | Height (cm) | Body mass (kg) | CMJ (cm) |
|  |  | Mean (sd) | Mean (sd) | Mean (sd) | Mean (sd) |  | Mean (sd) | Mean (sd) | Mean (sd) | Mean (sd) |
| 6-10 | 1193 | 9.8 (0.2) | 142.2 (6.0) | 34.7 (5.1) | 22.1 (3.4) | 171 | 9.9 (0.4) | 142.2 (6.2) | 35.1 (5.7) | 21.4 (4.0) |
| 11-15 | 21485 | 13.1 (1.2) | 161.5 (11.7) | 50.1 (11.6) | 27.3 (5.3) | 3493 | 13.2 (1.2) | 159.7 (8.2) | 51.0 (10.1) | 24.6 (4.0) |
| 16-20 | 1746 | 16.7 (0.9) | 179.3 (6.8) | 70.5 (8.8) | 35.3 (4.7) | 384 | 16.3 (0.5) | 166.1 (6.3) | 61.3 (8.8) | 26.9 (3.8) |

*Notes.* CMJ = Countermovement jump

Supplementary Table 3. Descriptive statistics of the TraDeRe dataset according to age groups and sex.

|  | **TraDeRe dataset – males** (n=142) | | | | | **TraDeRe – females** (n=184) | | | | |
| --- | --- | --- | --- | --- | --- | --- | --- | --- | --- | --- |
| Age group | Nr of subjects | Age (years) | Height (cm) | Body mass (kg) | CMJ (cm) | Nr of subjects | Age | Height (cm) | Body mass (kg) | CMJ (cm) |
|  |  | Mean (sd) | Mean (sd) | Mean (sd) | Mean (sd) |  | Mean (sd) | Mean (sd) | Mean (sd) | Mean (sd) |
| 16-20 | 2 | 19.5 (0.7) | 179.4 (9.2) | 64.0 (9.3) | 34.3 (4.8) | 2 | 19.5 (0.7) | 171.0 (4.2) | 56.9 (21.6) | 24.9 (8.9) |
| 21-25 | 14 | 23.3 (1.3) | 181.0 (7.4) | 86.6 (16.5) | 23.7 (4.7) | 10 | 23.2 (1.3) | 167.6 (8.5) | 72.3 (19.0) | 19.3 (6.1) |
| 26-30 | 18 | 28.4 (1.7) | 179.3 (4.2) | 87.1 (19.8) | 29.3 (9.4) | 24 | 28.5 (1.5) | 164.9 (6.3) | 66.5 (10.0) | 19.6 (4.1) |
| 31-35 | 28 | 33.3 (1.5) | 177.7 (6.3) | 82.2 (11.9) | 27.0 (6.1) | 31 | 33.4 (1.3) | 164.7 (7.6) | 67.3 (11.7) | 20.1 (5.6) |
| 36-40 | 27 | 37.8 (1.6) | 176.6 (5.8) | 83.2 (13.0) | 27.4 (5.8) | 52 | 38.5 (1.5) | 168.1 (6.2) | 70.0 (14.1) | 18.9 (5.4) |
| 41-45 | 38 | 43.2 (1.3) | 180.7 (7.1) | 85.8 (13.9) | 26.2 (4.3) | 53 | 42.7 (1.4) | 164.8 (6.2) | 73.6 (12.1) | 18.1 (4.7) |
| 46-50 | 15 | 46.9 (1.1) | 178.3 (7.1) | 86.1 (14.3) | 23.5 (3.6) | 12 | 47.1 (0.8) | 166.0 (6.5) | 69.9 (12.8) | 17.4 (4.0) |

*Notes.* CMJ = Countermovement jump

Supplementary Table 4. Descriptive statistics of the ERMA/EsmiRs dataset according to age groups.

|  | **ERMA / EsmirRs dataset – females** (n=870) | | | | |
| --- | --- | --- | --- | --- | --- |
| Age group | Nr of subjects | Age (years) | Height (cm) | Body mass (kg) | CMJ (cm) |
|  |  | Mean (sd) | Mean (sd) | Mean (sd) | Mean (sd) |
| 46-50 | 361 | 49.7 (0.9) | 166.0 (5.7) | 69.7 (10.4) | 19.8 (4.6) |
| 51-55 | 497 | 53.1 (1.2) | 165.1 (5.7) | 69.2 (10.9) | 18.7 (3.9) |
| 56-60 | 12 | 57.1 (0.9) | 165.0 (6.1) | 70.3 (11.4) | 16.6 (3.7) |

*Notes.* CMJ = Countermovement jump.

Supplementary Table 5. Descriptive statistics of the FERTILE/ExHRT dataset according to age groups.

|  | **FERTILE/ExHRT dataset – females** (n=105) | | | | |
| --- | --- | --- | --- | --- | --- |
| Age group | Nr of subjects | Age (years) | Height (cm) | Body mass (kg) | CMJ (cm) |
|  |  | Mean (sd) | Mean (sd) | Mean (sd) | Mean (sd) |
| 26-30 | 11 | 29.9 (0.3) | 166.1 (4.3) | 67.0 (12.9) | 27.8 (5.3) |
| 31-35 | 20 | 32.8 (1.4) | 165.6 (5.1) | 67.4 (14.6) | 23.7 (6.5) |
| 36-40 | 30 | 38.2 (1.2) | 161.5 (5.8) | 68.6 (14.4) | 23.8 (4.7) |
| 61-65 | 32 | 62.9 (1.3) | 162.6 (5.0) | 69.9 (11.1) | 12.8 (3.5) |
| >65 | 12 | 66.3 (0.7) | 158.5 (6.9) | 62.3 (158.5) | 13.7 (3.3) |


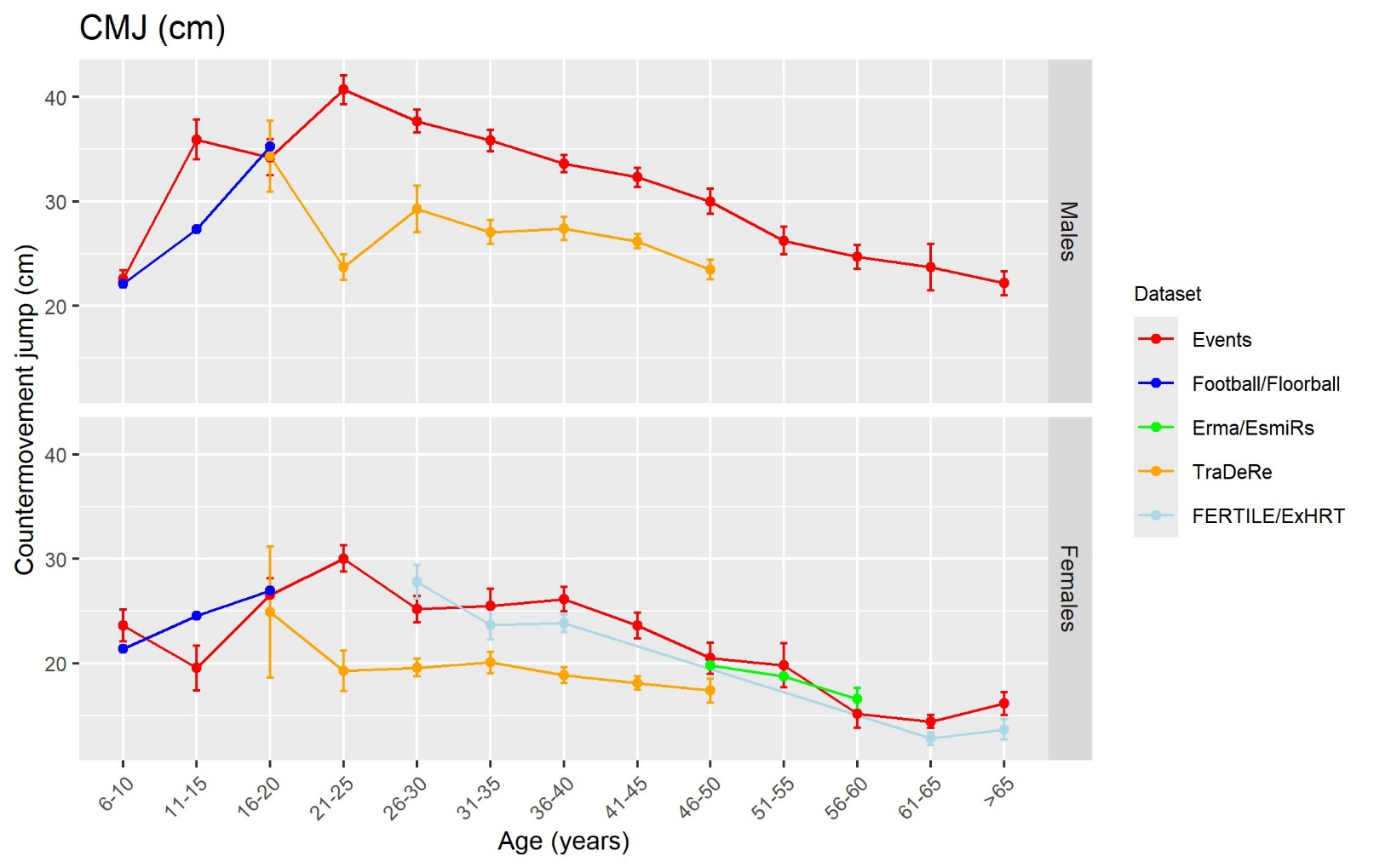


Supplementary Figure 1. The relationship between countermovement jump (CMJ) and age for males and females across four datasets. The plot displays the mean CMJ values (in cm) ± standard error (SE).


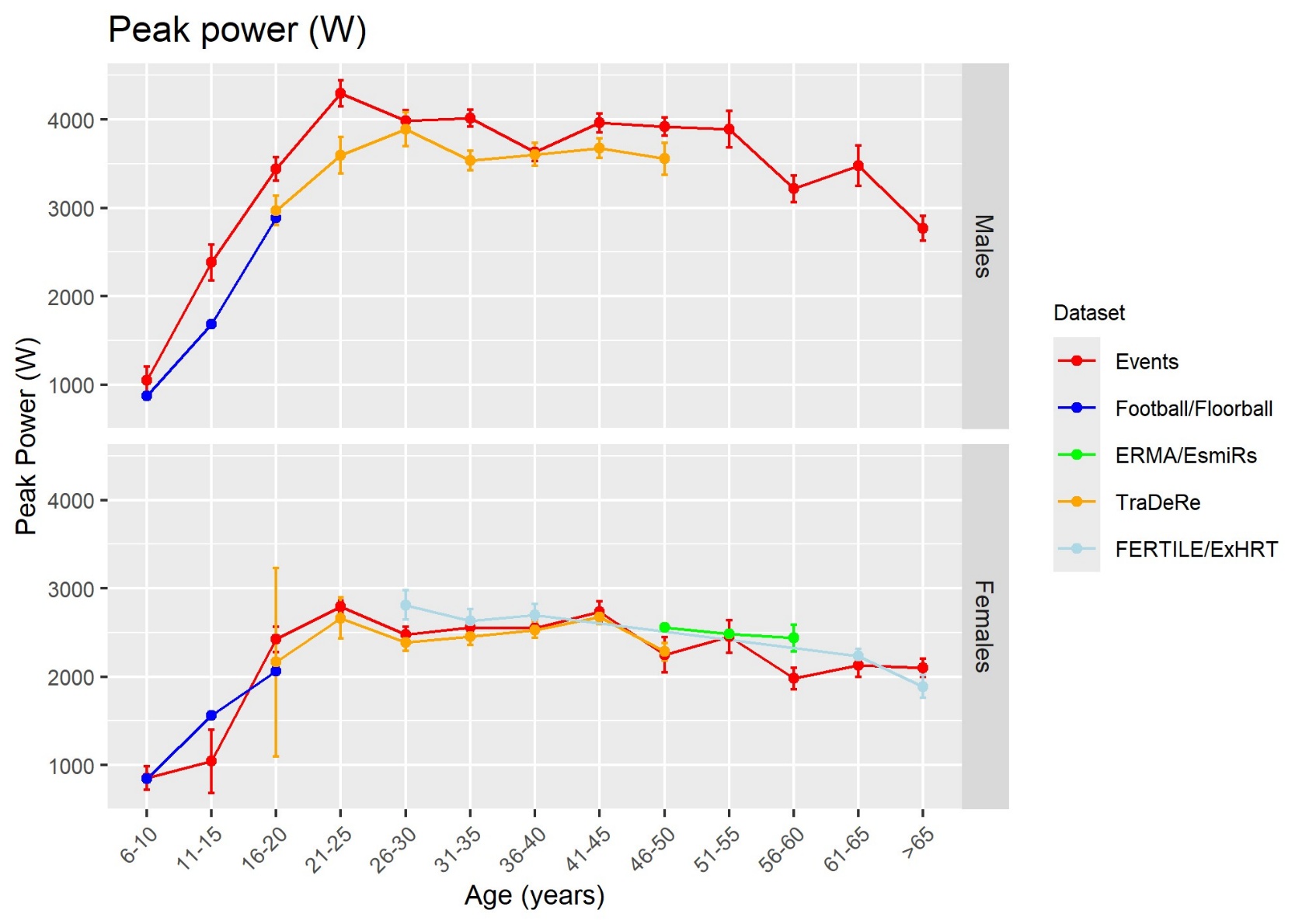


Supplementary Figure 2. The relationship between predicted peak power and age for males and females across five datasets. The plot displays the mean peak power values (W) ± standard errors (SE).


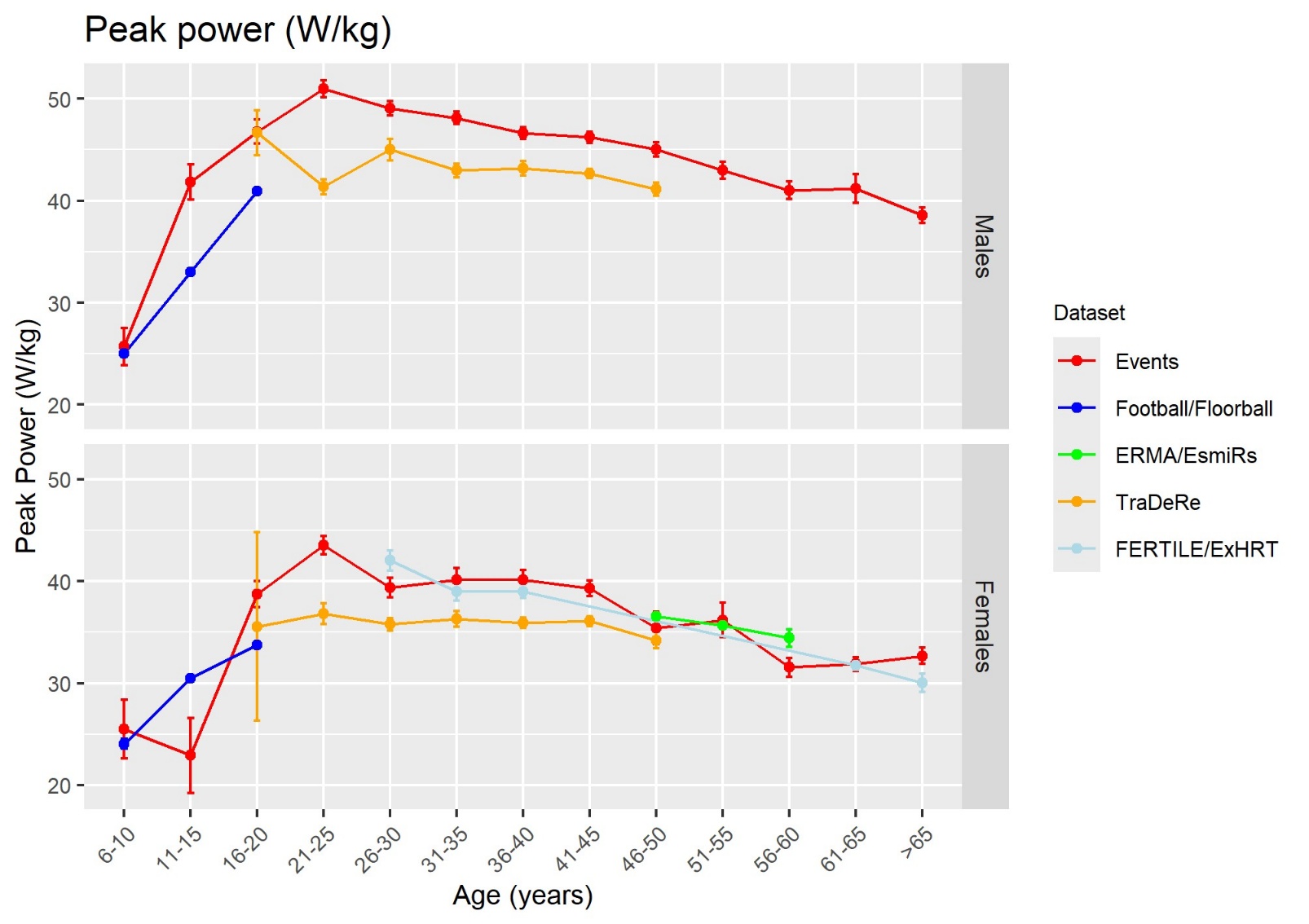


Supplementary Figure 3. The relationship between predicted peak power scaled to body mass and age for males and females across five datasets. The plot displays mean peak power values (W/kg) ± standard errors (SE).

Supplementary Table 6. Curvilinear regression analyses of the relationships between countermovement jump, peak power, and age.

|  |  | **Countermovement jump (cm)** | | | | **Peak power (W)** | | | | **Peak power (W/kg)** | | | |
| --- | --- | --- | --- | --- | --- | --- | --- | --- | --- | --- | --- | --- | --- |
| **Model** |  | **Males** |  | **Females** |  | **Males** |  | **Females** |  | **Males** |  | **Females** |  |
| Quadratic |  | B (SE) | *p* | B (SE) | *p* | B (SE) | *p* | B (SE) | *p* | B (SE) | *p* | B (SE) | *p* |
|  | Intercept | -1.32 (0.28) | **<.001** | 21.00 (0.33) | **<.001** | -2902.02 -2902.02 (26.03) | **<.001** | -52.45 (31.57) | **<.001** | -2902.02 -2.01 (0.30) | **<.001** | -17.41 (0.33) | **<.001** |
|  | Linear term | 2.76 (0.03) | **<.001** | 0.37 (0.03) | **<.001** | 427.95 (2.56) | **<.001** | 146.45 (2.80) | **<.001** | 3.30 (0.03) | **<.001** | 1.19 (0.03) | **<.001** |
|  | Quadratic term | -0.04 (0.00) | **<.001** | -0.01 (0.00) | **<.001** | -5.82 (0.05) | **<.001** | -1.85 (0.04) | **<.001** | -0.05 (0.00) | **<.001** | -0.02 (0.00) | **<.001** |
|  | R^2^ | 0.31 |  | 0.24 |  | 0.62 |  | 0.56 |  | 0.40 |  | 0.34 |  |
|  | AIC | 147 896 |  | 31 174 |  | 372 635 |  | 80 596 |  | 150 909 |  | 31 361 |  |
| Cubic |  |  |  |  |  |  |  |  |  |  |  |  |  |
|  | Intercept | -18.04 (0.47) | **<.001** | 11.44 (0.72) | **<.001** | –4574.00 (43.69) | **<.001** | -1654.00 (66.81) | **<.001** | -18.82 (0.51) | **<.001** | 4.31 (0.72) | **<.001** |
|  | Linear term | 5.17 (0.06) | **<.001** | 1.51 (0.08) | **<.001** | 668.20 (5.68) | **<.001** | 338.30 (7.64) | **<.001** | 5.69 (0.07) | **<.001** | 2.76 (0.0) | **<.001** |
|  | Quadratic term | -0.14 (0.00) | **<.001** | -0.04 (0.00) | **<.001** | -15.58 (0.22) | **<.001** | -7.91 (0.23) | **<.001** | -0.15 (0.00) | **<.001** | -0.07 (0.00) | **<.001** |
|  | Cubic term | 0.00 (0.00) | **<.001** | 0.00 (0.00) | **<.001** | 0.11 (0.00) | **<.001** | 0.06 (0.00) | **<.001** | 0.00 (0.00) | **<.001** | 0.00 (0.00) | **<.001** |
|  | R^2^ | 0.36 |  | 0.27 |  | 0.65 |  | 0.61 |  | 0.44 |  | 0.39 |  |
|  | AIC | 146 007 |  | 30 964 |  | 370 530 |  | 79 925 |  | 149 341 |  | 30 970 |  |
| Segmented | Intercept | -0.13 (0.23) | **<.001** | 13.09 (0.50) | **<.001** | -2455.40 (20.14) | **<.001** | -1067.27 (41.60) | **<.001** | 0.88 (0.24) | **<.001** | 11.48 (0.41) | **<.001** |
|  | Linear term (before breakpoint) | 2.10 (0.02) | **<.001** | 0.87 (0.04) | **<.001** | 316.47 (1.50) | **<.001** | 198.33 (3.04) | **<.001** | 2.44 (0.02) | **<.001** | 1.42 (0.03) | **<.001** |
|  | Change in slope after breakpoint | -2.43 (0.02) | **<.001** | -1.13 (0.04) | **<.001** | -338.89 (2.31) | **<.001** | -205.11 (3.59) | **<.001** | -2.68 (0.03) | **<.001** | -1.61 (0.04) | **<.001** |
|  | R^2^ | 0.38 |  | 0.30 |  | 0.67 |  | 0.63 |  | 0.44 |  | 0.40 |  |
|  | AIC | 145 181 |  | 30 777 |  | 369 229 |  | 79 665 |  | 149 067 |  | 30 873 |  |

Notes. B = unstandardized regression coefficient, SE = standard error, R^2^ adjusted (the variance explained by the model), AIC = Akaike Information Criterion. The linear term after the breakpoint represents the change in slope relative to the pre-breakpoint segment.
